# Supplementary material for: New Description of Evolution of Magnetic Phases in Artificial Honeycomb Lattice
Source: Sci Rep. 2017 Nov 22;7:16080. doi: 10.1038/s41598-017-15786-8 (PMC5700068; doi:10.1038/s41598-017-15786-8)
Supplement: Supplementary file 1 — Supplementary Materials [file 41598_2017_15786_MOESM1_ESM.pdf]

Supplementary Materials: New Description of Evolution of Magnetic Phases in Artificial Honeycomb Lattice

B. Summers[1,†], Y. Chen[1,†], A. Dahal[1], and D. K. Singh[1,*]
[1] *Department of Physics and Astronomy, University of Missouri, Columbia, MO 65211 and*
[*] *email: singhdk@missouri.edu*

Sample fabrication

The sample fabrication process utilizes diblock copolymer polystyrene(PS)-b-poly-4-vinyl pyridine (P4VP) of molecular weight 23k Dalton with the volume fraction of 70% PS and 30% P4VP. At this volume fraction, the diblock copolymer tends to self-assemble (under right condition) in a hexagonal cylindrical structure of P4VP in the matrix of polystyrene. A 0.5% PS-b-P4VP copolymer solution in toluene was placed in a heat bath at 60C for 1.5 hours and stored in a standard refrigerator. The solvent was spin coated onto cleaned silicon wafers at 2500 rpm for 30 s and placed in vacuum for 12 hours to dry. The samples were solvent annealed at 25o C for 12 hours in a mixture of THF/toluene (80:20 v/v) environment. The process results in the self-assembly of P4VP cylinders in a hexagonal pattern within a PS matrix, as shown schematically in step 1 of Fig. S1. Submerging the samples in ethanol for 20 minutes releases the P4VP cylinders yielding a porous hexagonal template (step 2, Fig.S1). Reactive ion etching with CF_4 gas was performed to transfer the hexagonal pattern to the silicon substrate (step3, Fig. S1). The top layer of the substrate resembles a honeycomb lattice pattern. This topographical property is exploited to create metallic honeycomb lattice by depositing permalloy, $Ni_{0.81}Fe_{0.19}$, in near parallel configuration in an electron-beam evaporation. For this purpose, a new sample holder was designed and setup inside the e-beam chamber. The substrate was rotated uniformly about its axis during the deposition to create uniformity. This allowed evaporated material to coat the top surface of the honeycomb only, producing the desired magnetic honeycomb lattice (step 4). Atomic force micrograph of a typical artificial honeycomb lattice is shown in Fig. 1a. Final thicknesses of permalloy film was $\simeq$ 7 nm.

Magnetic measurements

Magnetic measurements of permalloy artificial honeycomb lattice was performed using QD SQUID magnetometer. Magnetic field was applied in-plane to the sample. As shown in Fig. S2, four different magnetic regimes are indicated by dashed lines. The temperature dependent magnetization curves exhibit a tendency to attend the zero magnetization state at temperature below $T = 30$ K. This behavior becomes more apparent at higher magnetic field. For instance, at $H = 500$ Oe, the net magnetization of the honeycomb lattice reduces rapidly towards zero value at $T \leq 30$ K from the large saturation value. This behavior is only observed in the zero field cool measurement i.e. when the sample is cooled to the base temperature in zero magnetic field. Thus, the system develops the near zero magnetization state in the 'absence' of magnetic field, as expected in the spin solid phase of artificial honeycomb lattice. As soon as a magnetic field is applied, the correlated moments tend to abandon that delicate zero magnetization state. When cooled back in applied field (as small as $H = 25$ Oe), the moments remain locked in to the field-aligned value.

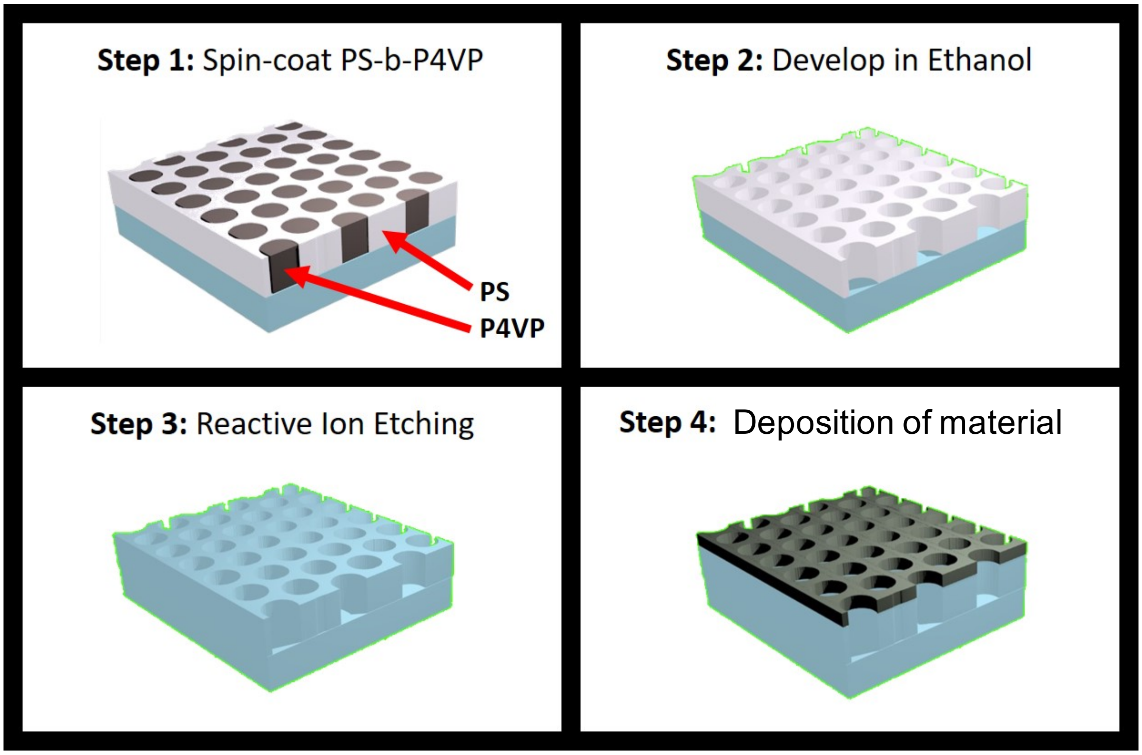

FIG. S1: **Step-by-step illustration of magnetic honeycomb lattice fabrication process, utilizing the self-assembly of diblock copolymer.** (a) The diblock film, PS-b-P4VP, after being spun onto Si wafer, dried in vacuum and annealed. (b) Developing the sample in ethanol cuts the ties between the two polymer and allows the P4VP cylinders to release from sample surface leaving PS matrix in honeycomb pattern. (c) RIE etches down into the substrate and removes most remaining polymer. (d) Using the near parallel deposition method in an electron-beam evaporator, we were able to deposit permalloy, $Ni_{0.81}Fe_{0.19}$, on top of the honeycomb pattern only. This produces the desired magnetic honeycomb system.

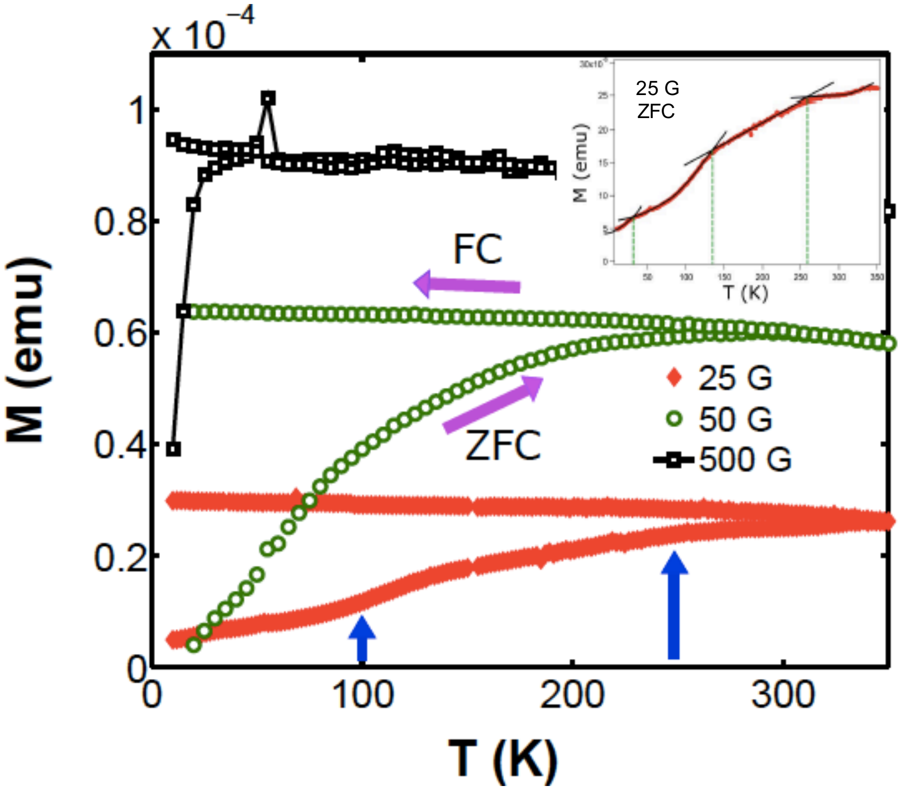

FIG. S2: **Magnetic measurements of permalloy artificial honeycomb lattice.** Zero field cool (ZFC) and field cool (FC) measurements at different field. Inset shows four possible magnetization regimes as a function of temperature.
